# The influence of natural environments on creativity

**DOI:** 10.3389/fpsyt.2022.895213

**Published:** 2022-07-27

**Authors:** Chin-Wen Yeh, Shih-Han Hung, Chun-Yen Chang

**Affiliations:** ^1^Department of Horticulture and Landscape Architecture, National Taiwan University, Taipei, Taiwan; ^2^Department of Landscape Architecture, Tunghai University, Taichung, Taiwan

**Keywords:** attention restoration, creative thinking, inspiration, naturalness, urban green space

## Abstract

This study investigated the effects of different natural environments on attention restoration and creativity. To compare the restorative benefits based on the degrees of perceived naturalness in urban areas, this study categorized environments into three types of perceived naturalness and tested the effect on one's creativity. The urban campus was selected as the study site, representing high-, medium-, and low-perceived naturalness photosets downloaded from Google Street Map images as experimental stimuli. The study invited 100 subjects to take the Abbreviated Torrance Test for Adults (ATTA), which measures creative thinking by viewing the onscreen photosets of the experimental stimuli. In addition, this study asked participants to complete the Perceived Restoration Scale (PRS) questionnaires. The results showed that high- and medium-perceived naturalness in the urban-campus site was superior to low-perceived naturalness in creative performance. In addition, there were significant differences in elaboration and flexibility for different degrees of perceived naturalness. Various degrees of perceived naturalness showed a substantial correlation between PRS scores and ATTA scores. The attention restoration benefits of high- and medium-naturalness environments improve creativity. Our study indicates that viewing natural environments stimulates curiosity and fosters flexibility and imagination, highly natural environments distract our minds from work, and the benefits of attention restoration can improve the uniqueness and diversity of creative ideas. This study provides a reference for creative environmental design and supports further understanding of nature's health and creativity benefits in urban areas.

## Introduction

Studies in environmental psychology have found that natural environments have psychological benefits, such as attention restoration theory (ART) (1) and stress reduction theory (SRT) (2), that greatly enhance essential human health. Previous study found that the forest landscape elicits one's ability to provide more detailed answers than an urban street landscape (3). However, most studies have focused on the psychological benefits of natural environments for self-actualization rather than for creativity. Exploring the degree of naturalness in urban green spaces would help us understand the effect on creative thinking and to understand the benefits of restorativeness.

Past research has shown that natural environments, or environments with natural elements, enhance creative performance more than urban environments (4–7). Dealing with the daily work process and preparing and understanding new work problems could consume our directed attention, leading to attention fatigue. Exposure to environments with restorative characteristics (i.e., being away, fascination, extent, compatibility) compared with artificial environments can promote recovery from attention fatigue (1). In addition, opportunistic assimilation theory says that the visual environment may stimulate inspiration and encourage creative thinking (8, 9), and nature characteristics such as bio-inspired, fascination attributes enhance one's creativity ability (10, 11). In addition, human perceived naturalness affects one's visual quality of the spaces (12, 13). While little research has examined the influence of the degrees of perceived naturalness in environments on creative performance, this research investigated whether environments with different perceived naturalness could impact creative performance. Therefore, this study explored the relationship between the different environments' attention-recovery benefits and the subjects' creative performance.

The research objectives were as follows:

1. Explore whether different degrees of natural environment in urban settings influence attention recovery.
2. Explore whether different degrees of natural environment in urban settings have an impact on creative performance.

### What is creativity?

Creativity refers to generating new and valuable ideas, identifying problems, and realizing ideas (11, 14, 15). To be innovative, ideas must be suitable for solving problems. Creativity is a process of generating ideas to manifest a problem-solving ability (16, 17) through complex cognitive methods of identifying questions, developing ideas, and then implementing the ideas. When individuals face work problems, one way to seek solutions is through divergent thinking (18). Divergent thinking means proposing many possible solutions to a problem and choosing the best solution, rather than seeking a single answer. Divergent thinking reflects many aspects of creativity and could be an essential indicator of knowing one's creative potential (16, 19). Guilford (18) pointed out that divergent thinking has four elements: fluency, originality, elaboration, and flexibility. Fluency is the ability to come up with many ideas and represents an individual's ability to recall past information widely and freely. Originality means combining different types of information to come up with innovative and unique ideas. Elaboration is the ability to express the details of an idea accurately and completely. Flexibility refers to multiple aspects of flexible thinking in response to the same stimuli. In addition, scholars have noted that the creative process has four stages: preparation, incubation, idea generation, and evaluation (14, 15). The incubation period occurs when a solution cannot be identified after sufficient preparation. The creator temporarily stops consciously thinking about the problem and directs their attention to other things. After a short rest, thoughts may flow freely in the subconscious without being restricted by general logic, generating new ideas more efficiently (14, 15).

When seeking inspiration for problem-solving, the inspiration may occur as a momentary “Ah-ha!” moment or gut reaction (20). More complex problems may require more directional attention on mentally consuming thinking. Sio and Ormerod (21) believed that fatigue recovery and external stimuli could affect the incubation factors. Fatigue recovery refers to the improvement of incubation benefits after recovering from mental fatigue when the initial tasks of understanding and thinking cannot solve the problem (22). On the other hand, opportunistic assimilation theory points out that the existing resources from external stimuli (i.e., information received from the external environment) can be used as inspiration to develop ideal solutions and bring the opportunity to inspire new ideas without subjects being aware of them and with no need for attention (23).

### Creativity, naturalness, and the restorative environment

Naturalness is defined as a biosphere with any type of natural element in the space. The degree of closeness to nature, such as nearness to water, plants, and other natural elements by visual perceptions, called perceived naturalness, might influence our landscape preference, restorative experience, and landscape design (12, 13, 24). In addition, information in nature stimulates ideas. In environmental design, such as landscape and architecture, biomimicry, inspired by the design method of technology transfer between biological and man-made structures in nature, is widely used in sustainable environmental design (25). Moreover, spaciousness and mystery in the natural environment elicit one's creativity performance (26).

A restorative environment provides a sense of escape from the usual, recovery from attention fatigue, and the potential to generate ideas through mind-wandering (e.g., daydreaming or freely thinking) (15). Studies point out that a natural setting with “fascination” stimulates ideas and influences creative ability, which is associated with mind-wandering (27). That is to say, natural environments easily attract involuntary attention, allowing the mind to roam freely and recover directed attention (1). The four restorative environmental characteristics bring out the conceptual statement mentioned in ART, which includes the following. Being Away, escape from everyday life, means presenting the ego with something different from everyday life. Extent, the range of visual perceptions in a wilderness environment, is vast, and such rich, diverse environmental information provides opportunities for visual exploration in comfortable, easy-to-read ways. In addition, the concept of extent includes connectedness and scope, which gives a sense of being whole (1). Nature is rich in fascinating elements that provide opportunities for “soft” Fascination, such as waterfalls, clouds, sunsets, snow scenes, or leaves fluttering in a breeze. The intriguing qualities of the natural environment can both attract involuntary attention and restore attention. Compatibility refers to the degree to which an individual's needs and tendencies are compatible with environmental conditions.

The most important influence of the natural environment on creativity is at the creative idea stage (i.e., in the incubation period), including getting inspiration and problem-solving (7, 11). Nature beneficially enhances creativity, new ideas, and flexible thinking, while improving our attention to analyze further and develop ideas (1, 3, 7, 11). During a creative incubation period, nature walks foster calmness and spiritual rejuvenation, providing opportunities to rest and review problematic issues in a new light (11). Numerous studies have discussed the relationship between creativity and natural environments, including actual nature experience (4, 28), indoor plants, natural window views or natural environmental images (3, 6, 8, 26, 29), natural environments experienced through immersive virtual devices (30), and even quick design practice in an actual outdoor natural environment (7). All the above studies found that creativity improves in natural settings or the presence of natural elements. Since creativity relies on the benefits that individuals obtain from environmental perceptions, the degree of perceived naturalness in this study mainly refers to the proportion of natural elements in the visual environment and how close to nature individuals judge the environment to be.

Based on these statements, this study aimed to explore the effects of different degrees of perceived naturalness in the urban environment on attention restoration and creativity. First, does the proportion of natural elements in an image affect attention restoration? Second, is creative performance affected by perceived environmental naturalness?

## Materials and methods

The Research Ethics Committee of the National Taiwan University approved this study (approval number: 202103HS033). The campus of National Taiwan University, which was selected as the research site, could be regarded as a small prototype of urban greenery. Using the ArcGIS software's fishnet tool, we set sampling points every 60 meters (about half a minute's walking distance) in the research area. We collected the latitude and longitude coordinates of 498 sampling points. Google Street Map images were downloaded for each sampling point to build a database of environmental photos. Operational photo parameters include viewing angle parallel to the line of sight, uniform, and the photo size (200 pixels by 200 pixels in download by the free version). After excluding 13 sampling points with no associated photos, a total of 485 street-view environmental photos remained. Next, reviewing these photos and eliminating those with unacceptable compositions (such as dark, distorted, or blurred images that might affect visual perception or photos with excessive repetition of similar scenery), 141 photos remained.

### Selected different degrees of perceived naturalness

We used the definition of perceived naturalness within the criteria shown in Table 1. We then divided the research stimuli into high-, medium-, and low-perceived naturalness in urban environments. The researchers selected 20 representative environmental photos for each group (i.e., 60 photos) from the 141 remaining photos. Five professional landscape architecture researchers were invited to evaluate the photos to verify them, with an inter-rater reliability statistic of 0.88 (*p* < 0.001). The evaluations of perceived naturalness were based on the statement, “I think the environment of this place is very natural.” They rated the perceived naturalness on a seven-point Likert scale (1 = strongly disagree, 7 = strongly agree). Finally, all the photos were classified as high-perceived naturalness (*M* = 5.20, *SD* < 0.35), medium-perceived naturalness (*M* = 3.74, *SD* < 0.47), and low-perceived naturalness (*M* = 1.64, *SD* < 0.56) environments in three experimental groups. There were 18 photos in each group, making a total of 54 photos (samples shown in Table 1).

**Table 1:** Definitions of environmental photo content for each degree of perceived naturalness.

**Group**	**Sample picture**	**Description**
High naturalness (HN)	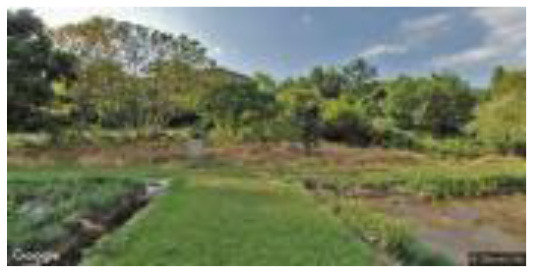	•Proximity to nature—very close to nature.•Proportion of natural elements—mainly natural elements, including flowers, trees and ground cover, with few artificial features such as trails and guardrails.
	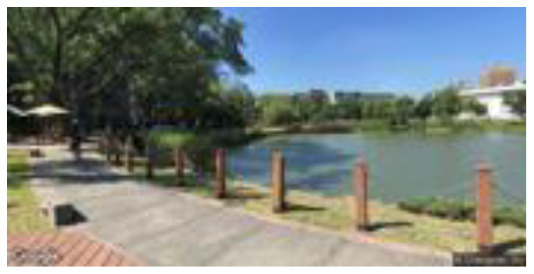	•Environment type—campus ecological pool, farmland, or green recreational area.
	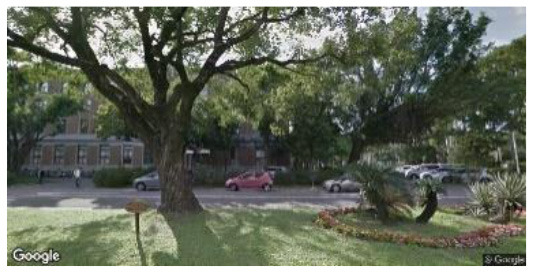	
Medium naturalness(MN)	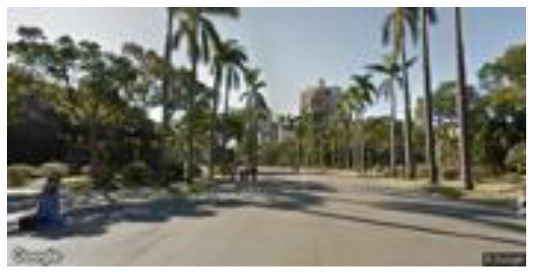	•Proximity to nature—a moderately close-to-nature semi-artificial and semi-natural environment. •Proportion of natural elements—a balance of natural elements and artificial elements, with artificially designed streets and planting configurations.
	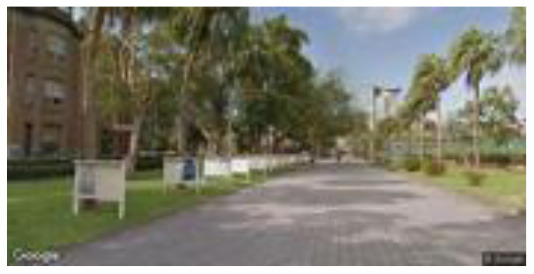	•Environment type—campus streets and outdoor recreation spaces.
	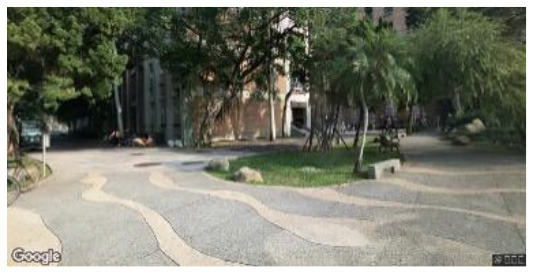	
Low naturalness (LN)	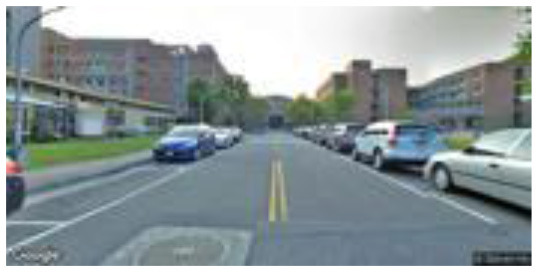	•Proximity to nature—very unnatural man-made environment.
	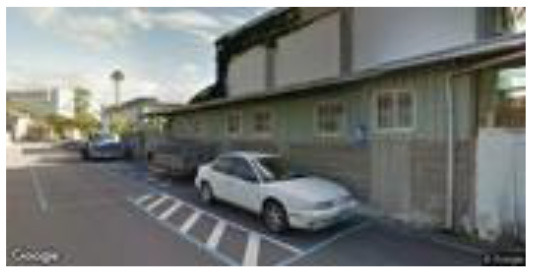	•Proportion of natural elements—a street environment with few natural elements, dominated by man-made facilities such as cars, buildings, and driveways.•Environment type—artificial environments such as campus parking lots and roads.
	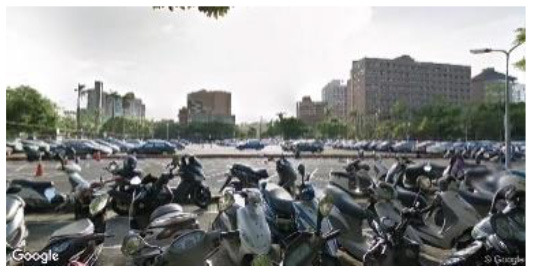	

### Perceived restoration scale

As reviewing numerous studies, studies did not specifically mention the order effect as discussing the restorativeness in different environments (31–34), so as in the short version of PRS developed by Berto (35). Based on the ART and the relationship between the natural environment and creativity ability, this study used the short version of the PRS developed by Berto (35) to measure restorative characteristics in different degrees of perceived naturalness in the urban settings. In addition, our study used the same order to test what subjects feel restorativeness in different naturalness urban.

Using this version, we translated the original text by researchers and discussed with native speakers the meaning of sentences accurately. Five items represented the distance from daily life and attractiveness in the environment: being away, fascination, coherence, scope, and compatibility. In ART, extent refers to a sense of coherence to the place and feeling a sense of scope in exploring the settings (1). Therefore, this study used the concepts of coherence and scope to measure restorativeness. Each item was scored on a five-point Likert scale (1 = strongly disagree, 5 = strongly agree). The study used average scores on each restorative characteristic and average total scores on the PRS to verify our research.

### Measurement of creative performance

#### The abbreviated Torrance test for adults

The ATTA measures creative performance. The test generates quantitative data, normative data, and valid standardized scores (16, 36). The purpose of the test was to determine the association between divergent thinking and stimuli during the creative thinking process of subjects and to provide an index to evaluate creative performance in a specific state. The study used the version that Taiwan Psychological Press published (https://www.psy.com.tw/ec99/ushop20128/GoodsDescr.asp?category_id=119&parent_id=87&prod_id=84150), which could be tested from February 2020 to January 2022.

According to the order of the experiment in the instruction manual, it consisted of three activities—one verbal activity and followed up with two figural activities published by Taiwan Psychological Press. Consequences tasks were used in the verbal activity test by asking participants to answer the hypothetical situations presented by the questions within a time limit. This type of test is similar to that of Hass (37). In Figural Activity 1, the subjects were asked to use the fragments of geometric figures provided in the questions to finish the drawing within a given time frame. In Figural Activity 2, the subjects were asked to use the nine identical graphics on the test paper to draw pictures while naming each sketch they drew.

#### Scoring of ATTA

The creativity evaluation method used Guilford's (18) four divergent thinking concepts for evaluation scoring criteria—fluency, originality, elaboration, and flexibility. In scoring fluency, people who come up with many ideas or solutions have the fluency ability of creativity. We used the number of those ideas as the scoring standard without incorrect answers. The original score in fluency is one and up to twenty-two. Originality stands for creating unusual, new, and unique ideas. The original score in originality is zero and up to seven. The elaboration scored as what participants presented the details, but not just the core idea. The original score in elaboration is zero and up to thirteen. Flexible thinking means creating more satisfactory answers as reposing to the same situation. Therefore, the original score in flexible is zero up to six. Those four indicators of creativity were scored as “original scores” based on the Torrance Creativity Test Instruction Manual (36). After scoring the four original scores, the scores were converted to a “nine-point normalized score”, from eleven to nineteen, conducive to comparison and discussion of the four indicators. The total normalized score (A) of the four indicators, represented creative performance.

In addition, the statistical analysis of the original scores of each activity and the total normalized score of creativity were then tested for the effect of different perceived naturalness. Another scoring system to verify one's details on creativity is called the “standard reference scores (B)” for 0, 1, and 2 points within each of the 15 indexes, such as the abilities of imagination, novelty, abstraction, feelings, stories, or fantasy and then added those points together.

Finally, the Creativity Index (CI) score is an overall concept of creativity, which distinguish the different level of creativity. In addition, the CI refers to the holistic ability to analyze one's creativity in performance. It was calculated by the total normalized score (A) and the standard reference score (B). The CI score from one to seventy-six or more.

**Figure 1:**
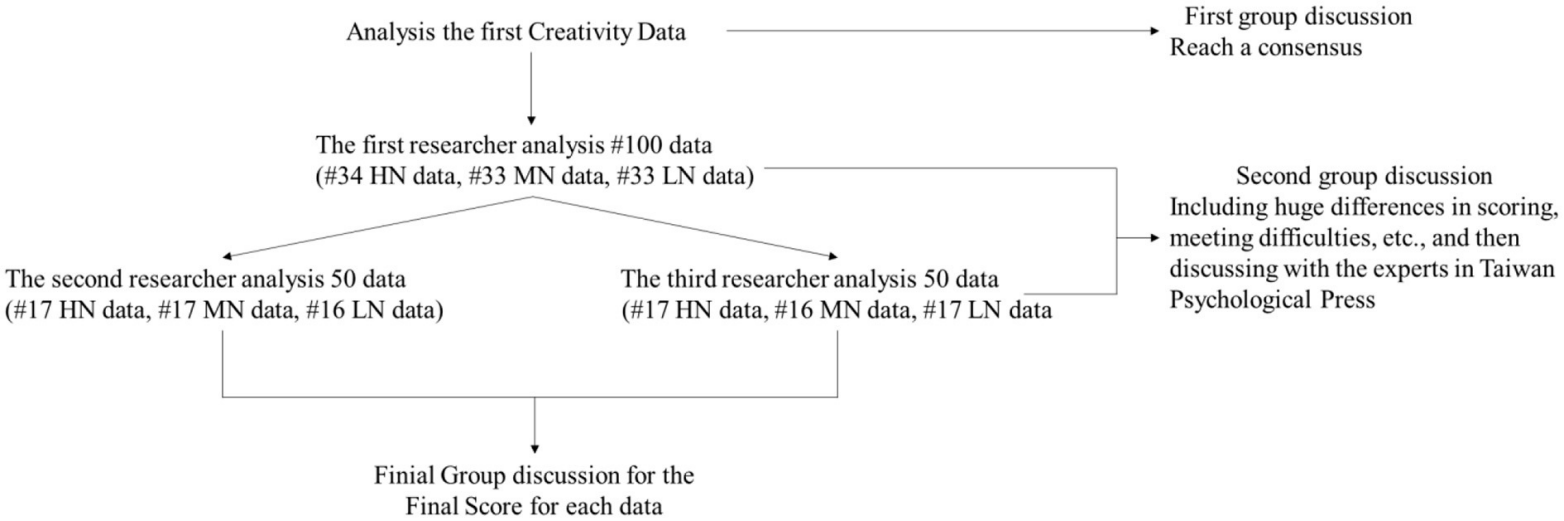
The scoring process among three researchers. High naturalness (HN), Medium naturalness (MN), Low naturalness (MN); # means numbers of data.

Further, three researchers were trained in the scoring method of the creativity test based on the instruction manual. In addition, the researchers had backgrounds in professional landscape environments and served as scorers. One researcher scored 100 test sheets and then invited two researchers to separately score in the second round. After that, the three researchers had a group discussion if there was a difference between the two researchers. In the meantime, researchers also included experts from Taiwan Psychological Press to discuss. After all this, the researchers reached a consensus and came up with the final score (Figure 1). Finally, after scoring all the data, they were sorted and entered statistical analysis software (Statistical Product and Service Solutions; SPSS) for subsequent analysis and discussion.

### Participants and procedure

**Figure 2:**
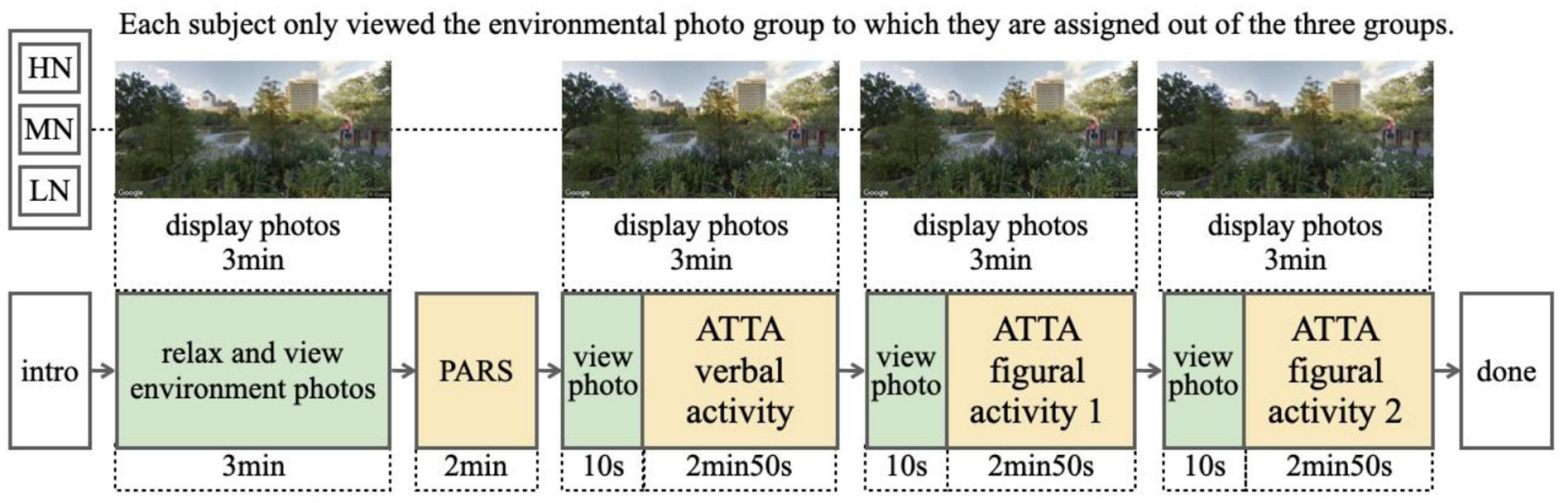
Experiment process.

One hundred subjects (male = 46; female = 54) were recruited online and divided into three experimental groups for high-, medium-, and low-perceived naturalness. Students over 20 years old were invited to participate in the study. The mean age was 23.1 years old. The subjects were randomly assigned to three experimental groups to view one of the photosets in the testing room for data collection. The experimental data collection included perceived attention restoration and creative performance. Attention restoration was measured using the PRS short-form questionnaire developed by Berto (35). Creativity was measured using the ATTA. The whole experiment process lasted about 30 min (Figure 2).

In more detail, each photo was displayed for 10 seconds, and each photoset of 18 photos was shown for 3 min (180 s). After viewing the photoset for the first time, the subjects completed the PRS. Next, the ATTA followed the second part of the experiment. In the meantime, as they participated in each creativity activity, the participants viewed the same photoset again. In addition, researchers told participants, “If you encounter any difficulties in the process, you can explore the environmental photos for inspirational clues for creative thinking.”

### Statistical approach

The study would like to test the effect of different degrees of perceived naturalness that influence one's creativity and restorativeness. Therefore, the study used Analysis of Variance (ANOVA) and Least Significant Difference (LSD) for the *post hoc* test to verify the research. The threshold for significance in *p* < 0.05 in our study. According to the creativity score, we used ANOVA to analysis on the “original score” and the “the overall ATTA normalized score”.

## Results

One hundred subjects participated in the experiment. One was excluded due to a lack of understanding of the content of the verbal activity, and two failed to answer the figural activity. Therefore, a total of 97 subjects were tested and separated to verify the effect of different degrees of perceived naturalness in an urban influence on one's creativity, while not missing data in testing the effect on perceived naturalness and restorativeness.

### The effect of different natural environments on restorativeness

**Figure 3:**
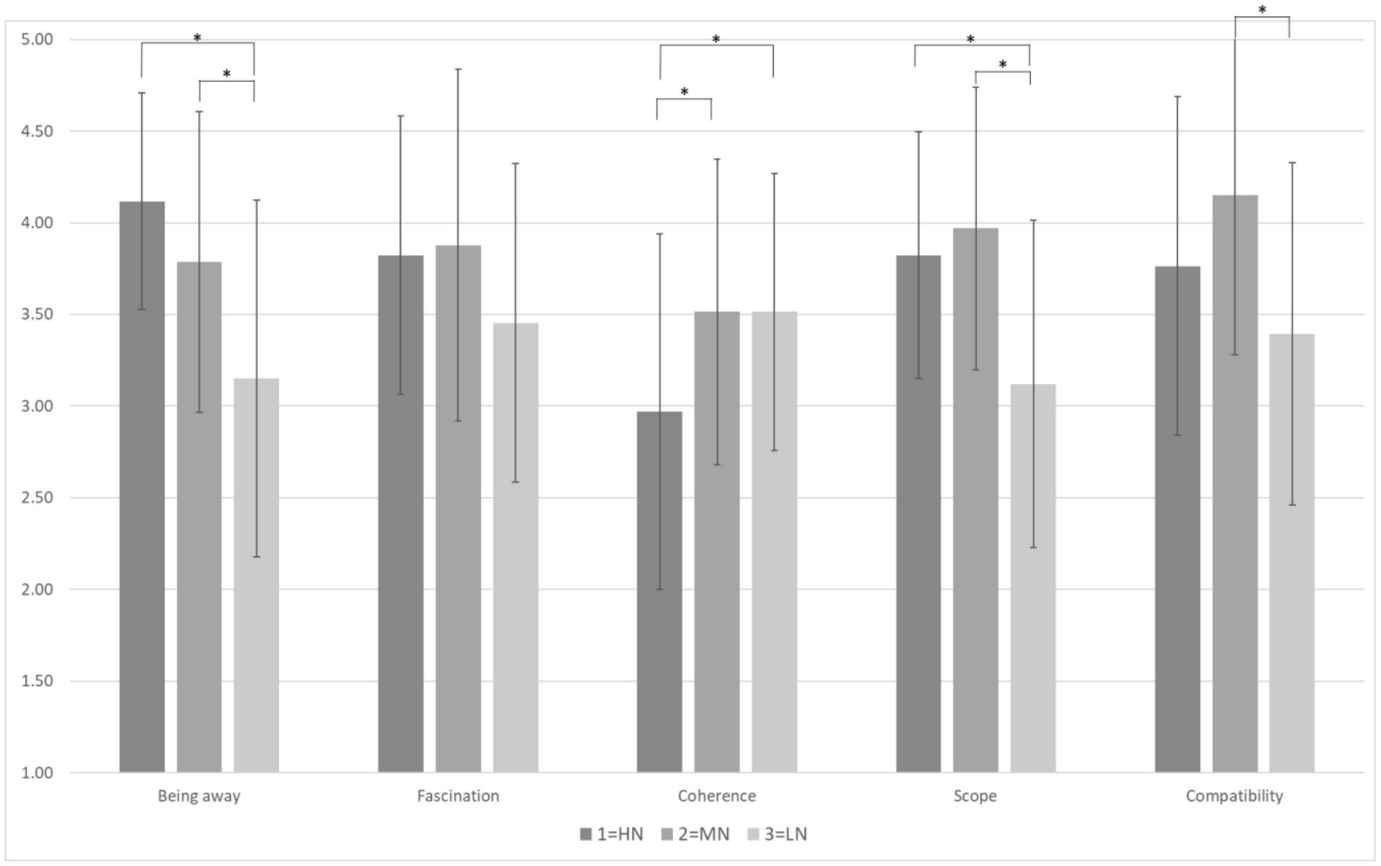
The effect of different degrees of perceived naturalness on restorativeness. *N* = *100, HN (n* = *34), MN (n* = *33), LN (n* = *33)*, *p< *0.05. This figure shows the five indicators of restorative of their means scores in different degrees of perceived naturalness. The error bars indicate the standard deviation*.

Table 2 shows significant differences in perceived naturalness and restorative characteristics. The results show the restorative characteristics of the PRS and show that the subjects who viewed the environments with different degrees of perceived naturalness had significantly different scores for being away [*F*_(2, 97)_ = 12.34, *p* < 0.001]. In the *post hoc* test, the HN group (*M* = 4.12, *SD* = 0.59) and the MN group (*M* = 3.79, *SD* = 0.82) were significantly higher than those in the LN group (*M* = 3.15, *SD* = 0.97). There was also a significant difference in scope [*F*_(2, 97)_ = 11.11, *p* < 0.001]. The *post hoc* showed the HN group (*M* = 3.82, *SD* = 0.67) and the MN group (*M* = 3.97, *SD* = 0.77) were significantly higher than for the LN group (*M* = 3.12, *SD* = 0.89). There was a significant effect on perceived naturalness and compatibility [*F*_(2, 97)_ = 5.73, *p* < 0.01]; the *post hoc* showed that the MN group (*M* = 4.15, *SD* = 0.87) was significantly higher than that of the LN group (*M* = 3.39, *SD* = 0.93). Moreover, there was a significant difference in the coherence dimension [*F*_(2, 97)_ = 4.52, *p* < 0.05]. The *post hoc* results showed that the MN group (*M* = 3.52, *SD* = 0.83) and those in the LN group (*M* = 3.52, *SD* = 0.76) had significantly higher scores than the HN group (*M* = 2.97, *SD* = 0.97). Figure 3 shows each restorative effect of different perceived naturalness in urban settings. The total PRS score was significant in different natural environments [*F*_(2, 97)_ =8.29, *p* < 0.05]. Through the *post hoc*, the results showed that the HN group (*M* = 18.5, *SD* = 2.29) and MN group (*M* = 19.30, *SD* = 2.89) both had a more significant restorativeness effect than those in the LN group (*M* = 16.64, *SD* = 2.98). The overall results aligned with Kaplan and Kaplan's ART (1), that is, environments with higher environmental perceived naturalness had the characteristics of recovering one's attention.

**Table 2:** One-way ANOVA analysis of PRS for environments with different degrees of perceived naturalness in urban settings.

	**HN (*****n** =* **34)**	**MN (*****n** =* **33)**	**LN (*****n** =* **33)**	**F**	**p**	**Post test**
	**M. (S.D.)**	**M. (S.D.)**	**M. (S.D.)**		
Being away	4.12 (0.59)	3.79 (0.82)	3.15 (0.97)	12.341	0.000	HN>LN* MN>LN*
Fascination	3.82 (0.76)	3.88 (0.96)	3.45 (0.87)	2.352	0.101	
Coherence	2.97 (0.97)	3.52 (0.83)	3.52 (0.76)	4.517	0.013	LN>HN* MN>HN*
Scope	3.82 (0.67)	3.97 (0.77)	3.12 (0.89)	11.111	0.000	HN>LN* MN>LN*
Compatibility	3.76 (0.92)	4.15 (0.87)	3.39 (0.93)	5.725	0.004	MN>LN*
PRS	18.50 (2.29)	19.30 (2.89)	16.64 (2.98)	8.293	0.000	HN>LN* MN>LN*

^*^p < 0.05.

The environment for the HN group included ecological pools, farmland, and other environments dominated by natural elements, whereas the environments for the MN and LN groups contained mainly artificial features. In addition, the main campus squares, sidewalks, driveways, and other environmental features were visually arranged more neatly and were more easily identified. Finally, the average score for the fascination dimension did not reach a significant level (*p* > 0.05). According to Kaplan and Kaplan (1), fascination is a attractive element or phenomenon in nature. In our study, the street scenes used in the research photos were familiar to most students and perhaps less appealing. It could be difficult for participants to feel a sense of attraction through the selected photos.

### The effect of different natural environments on creative performance

The ATTA consists of three activities. The original scores for each activity were first calculated during the scoring process, and the subjects' scores were compared with the Taiwan norm scores. We then tested the effects on different perceived naturalness. Creative thinking requires different abilities, depending on the type of activity. Therefore, the original scores used in each activity and the overall normalized score for ATTA creative performance was used to examine the effect on different naturalness.

**Figure 4:**
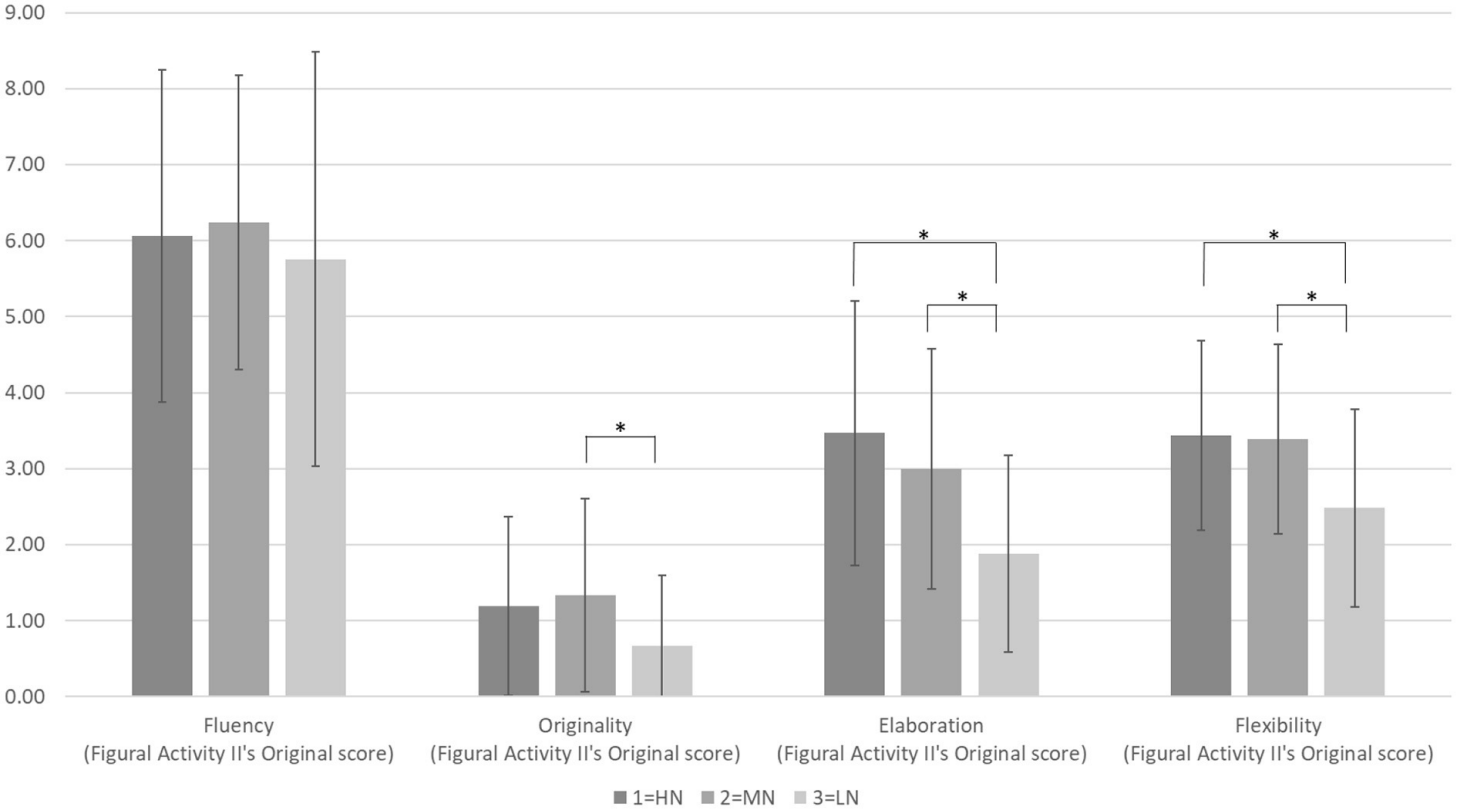
The effect of different degrees of perceived naturalness on creativity performance in Figural Activity 2. *N* = *98, HN (n* = *32), MN (n* = *33), LN (n* = *33)*, *p< *0.05. This figure shows the creativities indicators of their means scores in different degrees of perceived naturalness. The error bars indicate the standard deviation. Nineteen samples scored zero on “originality” in the low naturalness*.

The first activity was a verbal activity. There was no significant difference in the original scores for creativity for different degrees of perceived naturalness in the ability of fluency [(*F*_(2, 96)_ = 1.17, *p* = 0.32] and originality [*F*_(2, 96)_ = 1.68, *p* = 0.19]. The second activity was Figural Activity 1, for which the original score showed no significant effect on fluency [*F*_(2, 95)_ = 1.40, *p* = 0.25], originality [*F*_(2, 95)_ = 2.78, *p* = 0.76], and elaboration [*F*_(2, 95)_ = 1.25, *p* = 0.29] within different perceived naturalness. The third activity refers to Figural Activity 2. Table 3 and Figure 4 show the results of the original scores on originality [*F*_(2, 95)_ = 3.16, *p* < 0.05)], elaboration [*F*_(2, 95)_ = 9.10, *p* < 0.001)], and flexibility [*F*_(2, 95)_ = 5.93, *p* < 0.01)] and discovered a significant effect in different perceived naturalness. The *post hoc* test showed that originality in the MN group (*M* = 1.33, *SD* = 1.27) had a significantly higher score than the LN group (*M* = 0.67, *SD* = 0.92). For elaboration, the scores for the HN group (*M* = 3.47, *SD* = 1.74) and the MN group (*M* = 3.00, *SD*=1.58) were significantly higher than those for the LN group (M = 1.88, SD = 1.29). For flexibility, the HN group (*M* = 3.44, *SD* = *1.24*) and MN group (*M* = 3.39, *SD* = 1.25) had significantly higher scores than the LN group (*M* = 2.48, *SD* = 1.30). The fluency in this Figural Activity 2 was the only one that did not play a role among the perceived naturalness. The type of figural activity used in Studente et al. (6) study resembled Figural Activity 2 in our study. The experimental results for that study showed that the creativity score for the environment with natural elements was higher, consistent with this study's results for Figural Activity 2.

**Table 3:** One-way ANOVA analysis on the original score of ATTA's Figural Activity 2 in landscape environments with different perceived naturalness.

	**HN (*****n** =* **32)**	**MN (*****n** =* **33)**	**LN (*****n** =* **33)**	**F**	**p**	**Post test**
	**M. (S.D.)**	**M. (S.D.)**	**M. (S.D.)**			
Fluency	6.06 (2.18)	6.24 (1.94)	5.76 (2.73)	0.372	0.690	
Originality	1.19 (1.18)	1.33 (1.27)	0.67 (0.92)	3.162	0.047	MN>LN*
Elaboration	3.47 (1.74)	3.00 (1.58)	1.88 (1.29)	9.099	0.000	HN>LN* MN>LN*
Flexibility	3.44 (1.24)	3.39 (1.25)	2.48 (1.30)	5.932	0.004	HN>LN* MN>LN*

^*^p <0.05.

In addition, the study analyzed the effect on the overall creativity score in different degrees of perceived naturalness. Table 4 shows that there is a significant effect on the total normalized score on creativity and perceived naturalness [*F*_(2, 94)_ = 6.83, *p* < 0.01)]. The *post hoc* test showed that the HN group (*M* = 61.87, *SD* = 4.88) and the MN group (*M* = 61.61, SD = 5.14) had significantly higher scores than the LN group (*M* = 57.76, SD = 5.06). In addition, the effect on CI in different degrees of perceived naturalness showed significant differences [*F*_(2, 94)_ = 6.73, *p* < 0.01)]. The *post hoc* test showed that the HN group (*M* = 67.94, *SD* = 7.55) and the MN group (*M* = 67.33, *SD* = 7.20) had significantly higher scores than the LN group (*M* = 62.15, *SD* = 6.19). Considering the four dimensions separately, subjects viewing environments with different degrees of perceived naturalness showed significant differences in elaboration [*F*_(2, 94)_ = 4.51, *p* < 0.05)]. The results of the *post hoc* test showed that the HN (*M* = 16.26, *SD* = 1.55) and MN (*M* = 15.97, *SD* = 1.7) groups could elicit more elaboration ability in describing the activities than in the LN (*M* = 15.03, *SD* = 1.88). There was also a significant difference in flexibility [*F*_(2, 94)_ = 6.25, *p* < 0.01)]. The *post hoc* test showed that the HN group (*M* = 15.35, *SD* = 1.92) and the MN group (*M* = 15.15, *SD* = 2.09) had significantly higher scores than the LN group (*M* = 13.79, *SD* = 1.80). There is no significant effect on perceived naturalness and the creativity ability of fluency [*F*_(2, 94)_ = 1.77, *p* = 0.176)] and originality [*F*_(2, 94)_ = 2.86, *p* = 0.062)]. Overall, subjects viewing environments with high-perceived naturalness outperformed those viewing low-perceived naturalness environments in terms of creative performance scores, consistent with previous studies (6, 7, 26, 30).

**Table 4:** One-way ANOVA analysis of the overall ATTA normalized score of creativity for environments with different degrees of perceived naturalness.

	**HN (*****n** =* **31)**	**MN (*****n** =* **33)**	**LN (*****n** =* **33)**	**F**	**p**	**Post test**
	**M. (S.D.)**	**M. (S.D.)**	**M. (S.D.)**		
Fluency	15.26 (1.51)	15.39(1.35)	14.73 (1.68)	1.77	0.176	
Originality	15.00 (2.00)	15.12 (2.30)	14.00 (1.92)	2.86	0.062	
Elaboration	16.26 (1.55)	15.97 (1.70)	15.03 (1.88)	4.51	0.013	HN>LN* MN>LN*
Flexibility	15.35 (1.92)	15.15 (2.09)	13.79 (1.80)	6.25	0.003	HN>LN* MN>LN*
Total normalized score	61.87 (4.88)	61.61 (5.14)	57.76 (5.06)	6.83	0.002	HN>LN* MN>LN*
Creative index (CI)	67.94 (7.55)	67.33 (7.20)	62.15 (6.19)	6.73	0.002	HN>LN* MN>LN*

^*^p < 0.05.

## Discussion

Our study tests the effect of different perceived naturalness in urban environments on creativity and restorativeness using the ATTA and PRS scales. The results proved helpful in providing relevant suggestions and references for creative environmental design and confirming the importance of natural environments for creative performance.

### The findings on creative performance and different degrees of perceived naturalness in urban settings

The overall creativity scores showed a higher degree of perceived naturalness than lower, which elicits one's creativity ability from idea generation (flexibility) and the ability to express details (elaboration). There was no difference in verbal performance between the different perceived naturalness groups. According to the results, in terms of figural activity, our study inferred that those participants were students and familiar with the research sites with few changes of visual natural elements, which could be difficult for them to create many novelty ideas fluently. The results were in line with a previous study that stated there was no significant difference in the verbal performance with or without visual planting in the same classroom (6). In addition, there was no effect on Figural Activity 1. In Figural Activity 2, our results determined that regardless of high- or medium-perceived naturalness, urban environments with natural elements boost creative expression and, in particular, increase the ability for originality, elaboration, and flexibility more than in low-perceived naturalness environments. The results indicate that Figural Activity 2, with several identical graphics, fosters a sense of familiarity and good and creates ideas more than Figural Activity I. With a better understanding of the activity, the number of creative ideas gradually increases (38).

Based on past research and related theories on viewing images beforehand (39), unconsciously noticing text hints during quiz solving (23), and scanning the surrounding environment for clues during the creative process (9), it is evident that the surrounding environment can provide useful hints for the creative process and support creative thinking. The elements, compositions, and even symbolic meanings of natural environments provide viewers with space for free exploration and imagination, which are important for generating new ideas (40). Our findings showed that perceived naturalness does play a role in one's ability to describe details, which was consistent with a previous study's findings that forest environments contribute to an ability for sophistication (3).

### The findings on creative performance and restorativeness in different degrees of perceived naturalness in urban settings

This study consistently verified that the effects of different natural environments on attention restoration differ significantly. Environments with more natural elements have better perceived attention-restoration benefits than environments without natural elements. The interesting results of our findings were as follows. Being away, coherence, and scope correlated with creative performance in different degrees of perceived naturalness. High-perceived naturalness provided a sense of being away, significantly negatively related to elaboration. On the other hand, medium-perceived naturalness offers a sense of coherence and scope, which was significantly positively correlated to flexibility. A study found that the spaciousness of the natural environment positively affects creativity (11, 26). We might infer that on the psychological level, spaciousness is related to the search for innovative ideas and enables our minds to explore freely in the environment, especially for the extension to develop ideas. Being away was also highly correlated with perceived personal inspiration, suggesting that we are more motivated to think creatively when we feel disconnected from everyday life while it might limit the ability to describe details. In addition, creative performance is particularly highly correlated with flexibility, showing that natural environments away from the familiar allow us to be psychologically free and comfortable, thereby stimulating flexibility and generating diverse ideas. Therefore, our findings pointed out that the sense of coherence and scope in medium-perceived naturalness in urban settings might foster the flexibility of the creative thinking process and come up with various ideas. However, there was no significant effect on low-perceived naturalness between restorativeness and creativity.

### Limitation and future study

In this study, the urban-campus environment was used as the experimental stimulus, and the perceived naturalness of the environment was used to examine its influence on creativity. The perceived naturalness of environments has a positive impact on creativity. While there are limitations in our study. The “originality” could be hard for one to elicit “novelty” ideas through different naturalness, especially in urban green space. Therefore, it might cause affect normal distribution. Future research could test the different landscape types to find out the which kinds of landscape inspire our unusual thinking. In addition, the research results can help design environments that support psychological recovery, and confer creativity benefits, serving as a reference for environmental design. Natural elements not only provide psychological recovery benefits but also enhance creative performance. However, the effect of naturalness and restorativeness on creativity or the correlation between restorativeness and creativity performance is out of our scope of this study. Future research shall think about the research topic from the perspective of cognitive psychology and also from neuroscience. Those might go deeper and find the mechanism of connecting the brain and mind to explain the psychological state and creativity performance.

## Conclusion

The amount of visual natural landscape elements will affect people's perception of the perceived naturalness of the environment and thus affect the individual's creative ability and restorativeness. Past research has focused on the benefits of nature for creative performance or inspiration. As work fatigue accumulates, the number of creative ideas generated decreases (38), suggesting that restorative environmental characteristics are equally crucial for creative performance. This study indicates that viewing an environment with natural elements stimulates curiosity and a flexible imagination more than viewing an artificial environment. Also, natural environments allow our minds to temporarily detach from daily states, such as moments when we are in a daze or daydreaming, to obtain a “flash of inspiration.” More unique and diverse creative ideas become possible when opinions are flexible and multiple. Studies that explored the relationship between plant diversity, flower color diversity and richness, and perceived biodiversity showed that greater flower colors and diversity could attract visible species richness, lead to a more positive aesthetic experience, provide restoration benefits (41), and affect human health (42). Therefore, improving the perceived naturalness of urban landscapes by, for example, planting flowers might confer not only psychological benefits (43) but also enhance the natural elements and elicit creative thinking. Moreover, creating a “being away” and “scope” of restorative characteristics in urban settings could inspire one's creativity. Future research could analyze the types of natural elements, such as flowers, trees, rocks, and water, to verify how natural elements affect one's performance in creativity. With the increasing demand for creativity in future competitive environments, we look forward to creating healthier and more effective lifestyles through landscape environmental design.

## Data availability statement

The original contributions presented in the study are included in the article/Supplementary material, further inquiries can be directed to the corresponding author/s.

## Ethics statement

The studies involving human participants were reviewed and approved by the Research Ethics Committee of National Taiwan University (approval number: 202103HS033). The patients/participants provided their written informed consent to participate in this study.

## Author contributions

C-WY: conceptualization, methodology, formal analysis, investigation, validation, data curation, and writing-original draft. S-HH: conceptualization, validation, and writing-original draft. C-YC: conceptualization, validation, supervision, project administration, and funding acquisition. All authors contributed to the article and approved the submitted version.

## Funding

This study was supported by the Ministry of Science and Technology (109-2410-H-002−170 -MY2), Taiwan. The funder played no role in the study design, data collection, analysis, decision to publish, or preparation of the manuscript.

## Conflict of interest

The authors declare that the research was conducted in the absence of any commercial or financial relationships that could be construed as a potential conflict of interest.

## Publisher's note

All claims expressed in this article are solely those of the authors and do not necessarily represent those of their affiliated organizations, or those of the publisher, the editors and the reviewers. Any product that may be evaluated in this article, or claim that may be made by its manufacturer, is not guaranteed or endorsed by the publisher.
